# Has the COVID-19 Pandemic Affected the Epidemiology of Syphilis in Brazil?

**DOI:** 10.1055/s-0042-1748024

**Published:** 2022-05-24

**Authors:** Yago Tavares Pinheiro, Richardson Augusto Rosendo da Silva

**Affiliations:** 1 1 Department of Health Sciences, Postgraduate Program in Public Health, Federal University of Rio Grande do Norte, Natal, Rio Grande do Norte, Brazil

Dear Editor,

Syphilis is a sexually-transmitted infection (STI) identified as a public health problem in Brazil and worldwide. 1 According to the World Health Organization (WHO), 2 in 2016, 6.3 million cases of the disease were diagnosed worldwide, with an estimated global prevalence of 0.5%. In Brazil, the disease has come to be considered an epidemic due to the significant increase in the number of cases in recent years. 3

Syphilis is a disease of compulsory notification throughout the Brazilian territory. Data from the Ministry of Health indicate that, between 2010 and 2018, the incidence of syphilis in pregnant women increased from 3.5 to 21.4 cases per thousand live births. Regarding congenital syphilis, there was an increase from 2.4 to 9.0 cases per thousand live births. 4

In 2020, the problem of syphilis became even more serious due to the emergence of the coronavirus disease 2019 (COVID-19) pandemic, which changed the epidemiology of STIs worldwide. Some studies 5 6 7 8 performed in countries in Europe, Asia, North America, and the Caribbean have observed a reduction in the number of people diagnosed with syphilis during the pandemic, and this decrease was one of the consequences of social distancing measures and limited access to health services during this period. Moreover, Furlam et al. 9 reported that the COVID-19 pandemic generated a reduction in the number of syphilis diagnosis and treatment procedures in Brazil, in addition to the weakening of the relationship between users and the health system in the country. In this sense, it is essential to analyze how this context influenced the epidemiology of syphilis throughout Brazil.

We analyzed data from the Brazilian Notifiable Diseases Information System (Sistema de Informação de Agravos de Notificação, SINAN, in the Portuguese acronym) 10 of the Ministry of Health related to syphilis in pregnant women, and we observed a reduction of ∼ 1.1% in the total number of reported cases across Brazil between 2019 and 2020 (before and during the COVID-19 pandemic respectively). The Southern, Northeastern and Northern regions followed the national trend, showing reductions of around 5.3%, 4.7% and 0.5% respectively, of reported cases when comparing 2019 and 2020. On the other hand, in the same period, the Southeastern region showed an increase of 1.5% in reported cases, while in the Midwestern region there was stability ( Table 1 ).

**Table 1:** Reported cases of gestational syphilis and congenital syphilis in Brazil according to geographic region

Region	Reported cases of syphilis in pregnant women		Reported cases of congenital syphilis	
	2019	2020	2019	2020
Northern	6,120	6,092	2,232	1,808
Northeastern	13,197	12,585	6,523	6,232
Southeastern	28,113	28,558	10,869	9,883
Southern	9,486	8,987	3,267	2,973
Midwestern	5,180	5,180	1,464	1,240
Brazil	62,084	61,402	24,355	22,136

Data on congenital syphilis available on SINAN were also analyzed. We found a 9.2% reduction in reported cases of this condition between 2019 and 2020. Also, in the same period, all regions showed the same trend of reduction in reported cases, with the Northern and Midwestern being the regions that showed the highest rates of reduction (Northern: 19%, Midwestern: 15.4%, Southeastern: 9.1%, Southern: 9%, and Northeastern: 4.5%) ( Table 1 ). 10

Therefore, it has been noted that until 2019 the rates of syphilis in pregnant women and congenital syphilis showed a constant increase. However, from 2020 onwards, simultaneously with the emergence of the COVID-19 pandemic, these rates showed a downward trend. In this regard, we suggest that researchers develop more in-depth and systematized investigations that make it possible to understand the influence of the pandemic on the epidemiology of these infections in Brazil, so that it is possible to define whether the reduction is a consequence of social distancing measures and limited access to health services, or if it results from the effectiveness of disease-control strategies in the country.
